# Supplementary material for: Multigene Panel Germline Testing of 1333 Czech Patients with Ovarian Cancer
Source: Cancers (Basel). 2020 Apr 13;12(4):956. doi: 10.3390/cancers12040956 (PMC7226062; doi:10.3390/cancers12040956)
Supplement: Supplementary file 1 [file cancers-12-00956-s001.pdf]

Table S1. The clinicopathological characteristics of 1,333 ovarian cancer patients.

Category	N	% of known
Age at diagnosis (y)		
<30	84	6.46
30-39	152	11.69
40-49	282	21.69
50-59	360	27.69
60-69	328	25.23
>70	94	7.23
n.a.	33	
Personal history		
OC only	1045	78.39
OC & BC	210	15.75
OC & other cancer	78	5.85
Family history		
HOC	119	9.64
HBOC	345	27.96
Multiple cancer	182	14.75
None	588	47.65
n.a.	99	
Histology		
HG serous	478	42.68
LG serous	85	7.59
Serous unspecified	165	14.73
Endometrioid	90	8.04
Mucinous	43	3.84
Clear cell	15	1.34
Other	90	8.04
Border-line	154	13.75
n.a.	213	
Stage at diagnosis (FIGO)		
I	302	31.46
II	86	8.96
III	472	49.17
IV	100	10.42
n.a.	373	

Table S2. **Mutation frequencies in ovarian cancer cases and population-matched controls.**

Gene	1,333 OC patients N mutations (%)	1320 OC patients* N patients (%)	2,278 PMC N mutations (%)	OR (95% CI); p[a)]
Increased OC risk [b)]				
BRCA1 [c)]	**238 (17.85)**	**229 (17.35)**	**5 (0.22)**	**95.2 (40.1 - 295.2); 1.83×10^{-97}**
BRCA2 [c)]	**99 (7.43)**	**94 (7.12)**	**7 (0.31)**	**24.9 (11.6 - 63.6); 1.16×10^{-33}**
RAD51D	**13 (0.98)**	**13 (0.98)**	**2 (0.09)**	**11.3 (2.6 - 103.4); .9.66×10^{-5}**
RAD51C	**13 (0.98)**	**13 (0.98)**	**4 (0.18)**	**5.7 (1.7 - 23.8); 0.001**
BRIP1 [c)]	**11 (0.83)**	**10 (0.76)**	**5 (0.22)**	**3.5 (1.1 - 13); 0.03**
MLH1 [c)]	**5 (0.38)**	**4 (0.3)**	**1 (0.04)**	6.9 (0.7 - 340.4); 0.06 [d)]
MSH2	**3 (0.23)**	**3 (0.23)**	**0**	**- (-); 0.049** [d)]
MSH6	**3 (0.23)**	**3 (0.23)**	**0**	**- (-); 0.049** [d)]
STK11	2 (0.15)	2 (0.15)	0	- (-); 0.13
Potentially increase or insufficient evidence OC risk [b)]				
NBN [c)]	**16 (1.20)**	**14 (1.06)**	**7 (0.31)**	**3.5 (1.3 - 10.2); 0.006**
PALB2	8 (0.60)	8 (0.61)	9 (0.40)	1.5 (0.5 - 4.5); 0.45
ATM [c)]	7 (0.53)	6 (0.45)	8 (0.35)	1.3 (0.4 - 4.3); 0.78
BARD1 [c)]	**5 (0.38)**	**3 (0.23)**	**0**	**- (-); 0.049**
No increased risk of OC [b)]				
CHEK2 [c)]	13 (0.98)	11 (0.83)	8 (0.35)	2.4 (0.9 - 6.8); 0.06
TP53 [c)]	3 (0.23)	1 (0.08)	2 (0.09)	0.9 (0 - 16.6); 1
CDH1 [c)]	1 (0.08)	0	0	- (-); 1
PTEN [c)]	1 (0.08)	0	0	- (-); 1

* without multiple mutation carriers

[a)] OR, 95% CI and p was calculated in a group of 1320 OC patients following exclusion of 13 multiple mutation carriers (shown in Table S3).

[b)] Gene classification according to the NCCN guidelines version 2020.1. We found no mutations in *CDKN2A* and *NF1* in analyzed OC patients.

[c)] 13 multiple mutation carriers: 1×*BRCA1&ATM*, 1×*BRCA1&CHEK2*, 2×*BRCA1&NBN*, 1×*BRCA1&PTEN*, 2×*BRCA2&BARD1*, 1×*BRCA1&TP53,* 1×*BRCA1&BRCA2*, 1×*BRCA1&BRCA2&CDH1*, 1×*BRIP1&TP53*, 1×*BRCA1&MLH1*, 1×*BRCA2&CHEK2* (details provided in Table S3).

[d)] When analyzed Lynch syndrome genes collectively: OR=22.63 (95%CI 3.4 - 958.5); p=1.95×10^{-05}

Table S3. Clinical and pathological characteristics in multiple mutation carriers

Mutation 1	Mutation 2	Mutation 3	OC (age at dg; y)	BC (age at dg; y)	Other ca (age at dg; y)	Family history	OC Histology
BRCA1	*ATM*	-	46.7	49.8	melanoma (46)	HOC	HG serous
BRCA1	*CHEK2*	-	65.0	65.0	-	negative	HG serous
BRCA1	*NBN*	-	52.1	-	-	MCF	HG serous
BRCA1	*PTEN*	-	34	-	-	HBOC	HG serous
BRCA2	*BARD1*	-	58.6	-	-	HOC	HG serous
BRCA2	*BARD1*	-	53.0	-	-	HBOC	HG serous
BRCA1	*TP53*	-	40.8	-	-	HBOC	LG serous
BRCA1	*BRCA2*	-	48.7	46.5	-	HBOC	Other
BRIP1	*TP53*	-	30.0	44.0	-	MCF	Other
BRCA1	*BRCA2*	*CDH1*	56.0	n.a.	-	HBOC	n.a.
BRCA1	*MLH1*	-	42.3	38.3	endometrial (58)	HBOC	n.a.
BRCA1	*NBN*	-	46.0	-	-	HOC	n.a.
BRCA2	*CHEK2*	-	61.7	66.4	-	negative	n.a.

n.a. not available; HOC. hereditary ovarian cancer; HBOC. hereditary breast and ovarian cancer; MCF. Multiple cancer families; OC. ovarian cancer; BC. breast cancer; HG. high grade; LG. low grade

Table S4. Clinicopathological characteristics of mutation carriers in HBOC genes listed in NCCN guidelines (mutation carriers in a group of 1320 ovarian cancer patients (after exclusion of 13 multiple mutation carriers shown in Table S3). 10 genes significantly associated with OC risk in our study are highlighted.

	BRCA1	*BRCA2*	*RAD51C*	*RAD51D*	*BRIP1*	*MLH1*	*MSH2*	*MSH6*	*STK11*	*NBN*	*PALB2*	*ATM*	*BARD1*	*CHEK2*	*TP53*	*CDH1*	*PTEN*	*NF1*	**carriers*	*non-carriers*	*All patients without multiple mutation carriers*
Age at dg (y)																					
<30	2	1	2						1	1		1	1	4					13	71	84
30-39	20	1		1	0	1	2	1		1				1	0		0		28	122	150
40-49	79	12	4	3	2	1	1			2	2	1			0				107	171	278
50-59	84	38	5	5	4	1		1		6	1		1	3		0			149	207	356
60-69	32	33	2	4	3	1				3	3	3		2					86	240	326
>70	9	8			1			1	1	1	2	1	1	1	1				27	67	94
n.a.	3	1																	4	28	32
Personal history of cancer																					
OC & BC	73	28	2	1						4	4	1	1	2					116	87	203
OC only	149	60	10	12	10	1	2	2	2	9	4	5	1	9	1				277	761	1038
OC & non-BC	7	6	1			3	1	1		1			1						21	58	79
Family history of cancer																					
HOC	42	11	1				1	1					1	3					60	56	116
HBOC	90	37	5	1	2		2			1	1	1	1	2	1				144	195	339
MCF	33	14		1		1		1		3	2	2		2					59	121	180
no	57	28	5	10	7	3			1	9	5	3	1	4					133	454	587
n.a.	7	4	2	1	1			1	1	1									18	80	98
Stage at diagnosis (y)																					
I	26	12	1	1	1	3	1	2		7		1		3					58	241	299
II	20	2		2		1				2				2					29	57	86
III	109	39	6	8	4		1				3	1	2	4					177	289	466
IV	21	4	4		2					2	2	1			1				37	63	100
n.a.	53	37	2	2	3		1	1	2	3	3	3	1	2					113	256	369
Histology																					
HG serous	101	45	5	7	2	2	2			3	5	2	1	3					178	294	472
N.S. serous	39	13	1	3	2					1			1	1					61	104	165
Borderline	34	10	1	1	2					1		2			1				52	100	152
Endometrioid	16	2	2	1	1	2				2				4					30	60	90
Mucinous	3	2								2		1							8	35	43
LG serous	8	3	1																12	72	84
Clear cell	1							1											2	13	15
Other	2	1			1			1	1	2	1			1					10	80	90
n.a.	25	18	3	1	2		1	1	1	3	2	1	1	2					61	148	209
Sum	*229*	*94*	*13*	*13*	*10*	*4*	*3*	*3*	*2*	*14*	*8*	*6*	*3*	*11*	*1*	*0*	*0*	*0*	*414*	*906*	*1320*

Table S5. Mutations in 201 additional analyzed genes with associated OC risk. Significantly associated genes (p<0.05) are highlighted.

	All patients (N=1,333)		Controls (N=2,278)					
**Gene**	**N (Mut)**	**N (WT)**	**N (Mut)**	**N (WT)**	**OR**	**95% CI**		**p**
PPM1D	15	1318	2	2276	12.9	3.0	116.9	1.8E-05
NAT1	13	1320	5	2273	4.5	1.5	16.1	0.0027
FAN1	8	1325	6	2272	2.3	0.7	8.0	0.16
MMP8	6	1327	4	2274	2.6	0.6	12.4	0.19
PMS1	6	1327	3	2275	3.4	0.7	21.2	0.084
HELQ	6	1327	2	2276	5.1	0.9	52.2	0.058
FANCG	5	1328	2	2276	4.3	0.7	45.0	0.11
EXO1	5	1328	2	2276	4.3	0.7	45.0	0.11
RECQL4	5	1328	6	2272	1.4	0.3	5.6	0.55
FANCM	5	1328	6	2272	1.4	0.3	5.6	0.55
SHPRH	5	1328	1	2277	8.6	1.0	404.8	0.028
FANCI	5	1328	6	2272	1.4	0.3	5.6	0.55
RAD50	5	1328	5	2273	1.7	0.4	7.5	0.51
RECQL	4	1329	7	2271	1.0	0.2	3.8	1.00
RECQL5	4	1329	7	2271	1.0	0.2	3.8	1.00
MCPH1	4	1329	11	2267	0.6	0.1	2.1	0.59
RAD54L	4	1329	4	2274	1.7	0.3	9.2	0.48
FANCD2	3	1330	1	2277	5.1	0.4	269.3	0.15
MLH3	3	1330	3	2275	1.7	0.2	12.8	0.68
FANCA	3	1330	2	2276	2.6	0.3	30.8	0.37
SETX	3	1330	2	2276	2.6	0.3	30.8	0.37
TELO2	3	1330	4	2274	1.3	0.2	7.6	0.71
MSR1	3	1330	5	2273	1.0	0.2	5.3	1.00
MSH3	3	1330	9	2269	0.6	0.1	2.3	0.55
RFC4	3	1330	0	2278	n.a.			0.050
ZNF365	3	1330	0	2278	n.a.			0.050
MPL	3	1330	1	2277	5.1	0.4	269.3	0.15
RAD18	3	1330	1	2277	5.1	0.4	269.3	0.15
ALK	3	1330	3	2275	1.7	0.2	12.8	0.68
SLX4	3	1330	3	2275	1.7	0.2	12.8	0.68
MUS81	2	1331	1	2277	3.4	0.2	201.7	0.56
NF1	2	1331	1	2277	3.4	0.2	201.7	0.56
XPA	2	1331	1	2277	3.4	0.2	201.7	0.56
DPYD	2	1331	2	2276	1.7	0.1	23.6	0.63
RNF168	2	1331	2	2276	1.7	0.1	23.6	0.63
OGG1	2	1331	3	2275	1.1	0.1	10.0	1.00
RAD51B	2	1331	3	2275	1.1	0.1	10.0	1.00
TLR2	2	1331	3	2275	1.1	0.1	10.0	1.00
FANCF	2	1331	4	2274	0.9	0.1	6.0	1.00
ATR	2	1331	6	2272	0.6	0.1	3.2	0.72
WRN	2	1331	8	2270	0.4	0.0	2.1	0.34
BLM	2	1331	9	2269	0.4	0.0	1.8	0.35
XRCC1	2	1331	9	2269	0.4	0.0	1.8	0.35
BUB1B	2	1331	0	2278	n.a.			0.14
ERCC5	2	1331	0	2278	n.a.			0.14
RAD52	2	1331	0	2278	n.a.			0.14
BRCC3	2	1331	1	2277	3.4	0.2	201.7	0.56
UIMC1	2	1331	1	2277	3.4	0.2	201.7	0.56
PLA2G2A	2	1331	2	2276	1.7	0.1	23.6	0.63
POLE	2	1331	3	2275	1.1	0.1	10.0	1.00
ATRIP	2	1331	4	2274	0.9	0.1	6.0	1.00
DNAJC21	2	1331	4	2274	0.9	0.1	6.0	1.00
ERCC3	2	1331	10	2268	0.3	0.0	1.6	0.23
ERCC6	2	1331	0	2278	n.a.			0.14
FAAP24	2	1331	0	2278	n.a.			0.14
APEX1	2	1331	5	2273	0.7	0.1	4.2	1.00
CASP8	1	1332	1	2277	1.7	0.0	134.1	1.00
DMC1	1	1332	1	2277	1.7	0.0	134.1	1.00
FANCE	1	1332	1	2277	1.7	0.0	134.1	1.00
FLCN	1	1332	1	2277	1.7	0.0	134.1	1.00
RAD1	1	1332	1	2277	1.7	0.0	134.1	1.00
SDHAF2	1	1332	1	2277	1.7	0.0	134.1	1.00
XRCC4	1	1332	1	2277	1.7	0.0	134.1	1.00
CWF19L2	1	1332	2	2276	0.9	0.0	16.4	1.00
POLD1	1	1332	2	2276	0.9	0.0	16.4	1.00
TLR4	1	1332	2	2276	0.9	0.0	16.4	1.00
NCAM1	1	1332	3	2275	0.6	0.0	7.1	1.00
PRF1	1	1332	3	2275	0.6	0.0	7.1	1.00
TSHR	1	1332	3	2275	0.6	0.0	7.1	1.00
LIG3	1	1332	4	2274	0.4	0.0	4.3	0.66
RAD54B	1	1332	5	2273	0.3	0.0	3.1	0.42
DCLRE1C	1	1332	10	2268	0.2	0.0	1.2	0.064
MDC1	1	1332	13	2265	0.1	0.0	0.9	0.024
BRAP	1	1332	0	2278	n.a.			0.37
GRB7	1	1332	0	2278	n.a.			0.37
HOXB13	1	1332	0	2278	n.a.			0.37
CHEK1	1	1332	0	2278	n.a.			0.37
LIG4	1	1332	0	2278	n.a.			0.37
PTCH1	1	1332	0	2278	n.a.			0.37
RAD9A	1	1332	0	2278	n.a.			0.37
RUNX1	1	1332	0	2278	n.a.			0.37
SBDS	1	1332	0	2278	n.a.			0.37

TERF2	1	1332	0	2278	n.a.			0.37
AIP	1	1332	1	2277	1.7	0.0	134.1	1.00
FAM175A	1	1332	1	2277	1.7	0.0	134.1	1.00
EPHX1	1	1332	2	2276	0.9	0.0	16.4	1.00
RBBP8	1	1332	2	2276	0.9	0.0	16.4	1.00
ERCC2	1	1332	6	2272	0.3	0.0	2.3	0.27
RFC1	1	1332	0	2278	n.a.			0.37
ATMIN	0	1333	1	2277	0.0	0.0	66.6	1.00
BABAM1	0	1333	1	2277	0.0	0.0	66.6	1.00
BRE	0	1333	1	2277	0.0	0.0	66.6	1.00
CSNK1D	0	1333	1	2277	0.0	0.0	66.6	1.00
CYLD	0	1333	1	2277	0.0	0.0	66.6	1.00
DDB2	0	1333	1	2277	0.0	0.0	66.6	1.00
ERCC4	0	1333	1	2277	0.0	0.0	66.6	1.00
EXT2	0	1333	1	2277	0.0	0.0	66.6	1.00
EYA2	0	1333	1	2277	0.0	0.0	66.6	1.00
FANCL	0	1333	1	2277	0.0	0.0	66.6	1.00
HUS1	0	1333	1	2277	0.0	0.0	66.6	1.00
KCNJ5	0	1333	1	2277	0.0	0.0	66.6	1.00
NELFB	0	1333	1	2277	0.0	0.0	66.6	1.00
NFKBIZ	0	1333	1	2277	0.0	0.0	66.6	1.00
NSD1	0	1333	1	2277	0.0	0.0	66.6	1.00
POLB	0	1333	1	2277	0.0	0.0	66.6	1.00
PRKDC	0	1333	1	2277	0.0	0.0	66.6	1.00
PTTG2	0	1333	1	2277	0.0	0.0	66.6	1.00
RHBDF2	0	1333	1	2277	0.0	0.0	66.6	1.00
TCL1A	0	1333	1	2277	0.0	0.0	66.6	1.00
TP53BP1	0	1333	1	2277	0.0	0.0	66.6	1.00
TSC1	0	1333	1	2277	0.0	0.0	66.6	1.00
UBE2V2	0	1333	1	2277	0.0	0.0	66.6	1.00
XPC	0	1333	1	2277	0.0	0.0	66.6	1.00
ZNF350	0	1333	1	2277	0.0	0.0	66.6	1.00
CEP57	0	1333	2	2276	0.0	0.0	9.1	0.53
FANCB	0	1333	2	2276	0.0	0.0	9.1	0.53
MUTYH	0	1333	2	2276	0.0	0.0	9.1	0.53
RFC1	0	1333	2	2276	0.0	0.0	9.1	0.53
XRCC2	0	1333	2	2276	0.0	0.0	9.1	0.53
XRCC3	0	1333	2	2276	0.0	0.0	9.1	0.53
ESR2	0	1333	3	2275	0.0	0.0	4.1	0.30
HNF1A	0	1333	3	2275	0.0	0.0	4.1	0.30
MSH5	0	1333	3	2275	0.0	0.0	4.1	0.30
PREX2	0	1333	4	2274	0.0	0.0	2.6	0.30
FANCC	0	1333	5	2273	0.0	0.0	1.9	0.17
SMAD4	0	1333	5	2273	0.0	0.0	1.9	0.17
AURKA	0	1333	9	2269	0.0	0.0	0.9	0.031

Table 5. cont. Prevalence of mutations in 201 additional genes in 934 patients negative for mutations in 10 genes significantly associated with OC in our study

	Negative pts (N=934)*		Controls (N=2.278)					
	**N (Mut)**	**N (WT)**	**N (Mut)**	**N**	**OR**	**95% CI**		**p**
PPM1D	12	922	2	2276	14.8	3.3	136.7	1.7E-05
NAT1	8	926	5	2273	3.9	1.1	15.3	0.0262
FAN1	5	929	6	2272	2.0	0.5	8.0	0.32
MMP8	6	928	4	2274	3.7	0.9	17.7	0.041
PMS1	5	929	3	2275	4.1	0.8	26.3	0.051
HELQ	4	930	2	2276	4.9	0.7	54.1	0.063
FANCG	5	929	2	2276	6.1	1.0	64.4	0.025
EXO1	4	930	2	2276	4.9	0.7	54.1	0.063
RECQL4	4	930	6	2272	1.6	0.3	6.9	0.49
FANCM	3	931	6	2272	1.2	0.2	5.7	0.73
SHPRH	2	932	1	2277	4.9	0.3	287.9	0.20
FANCI	2	932	6	2272	0.8	0.1	4.6	1.00
RAD50	2	932	5	2273	1.0	0.1	6.0	1.00
RECQL	3	931	7	2271	1.0	0.2	4.6	1.00
RECQL5	3	931	7	2271	1.0	0.2	4.6	1.00
MCPH1	3	931	11	2267	0.7	0.1	2.5	0.77
RAD54L	2	932	4	2274	1.2	0.1	8.5	1.00
FANCD2	3	931	1	2277	7.3	0.6	384.5	0.077
MLH3	3	931	3	2275	2.4	0.3	18.3	0.37
FANCA	2	932	2	2276	2.4	0.2	33.7	0.58
SETX	2	932	2	2276	2.4	0.2	33.7	0.58
TELO2	2	932	4	2274	1.2	0.1	8.5	1.00
MSR1	2	932	5	2273	1.0	0.1	6.0	1.00
MSH3	2	932	9	2269	0.5	0.1	2.6	0.53
RFC4	2	932	0	2278	n.a.			0.084
ZNF365	2	932	0	2278	n.a.			0.084
MPL	1	933	1	2277	2.4	0.0	191.4	0.50
RAD18	1	933	1	2277	2.4	0.0	191.4	0.50
ALK	1	933	3	2275	0.8	0.0	10.1	1.00
SLX4	1	933	3	2275	0.8	0.0	10.1	1.00
MUS81	2	932	1	2277	4.9	0.3	287.9	0.20
NF1	2	932	1	2277	4.9	0.3	287.9	0.20
XPA	2	932	1	2277	4.9	0.3	287.9	0.20
DPYD	2	932	2	2276	2.4	0.2	33.7	0.58
RNF168	2	932	2	2276	2.4	0.2	33.7	0.58
OGG1	2	932	3	2275	1.6	0.1	14.2	0.63
RAD51B	2	932	3	2275	1.6	0.1	14.2	0.63

TLR2	2	932	3	2275	1.6	0.1	14.2	0.63
FANCF	2	932	4	2274	1.2	0.1	8.5	1.00
ATR	2	932	6	2272	0.8	0.1	4.6	1.00
WRN	2	932	8	2270	0.6	0.1	3.1	0.73
BLM	2	932	9	2269	0.5	0.1	2.6	0.53
XRCC1	2	932	9	2269	0.5	0.1	2.6	0.53
BUB1B	2	932	0	2278	n.a.			0.084
ERCC5	2	932	0	2278	n.a.			0.084
RAD52	2	932	0	2278	n.a.			0.084
BRCC3	1	933	1	2277	2.4	0.0	191.4	0.50
UIMC1	1	933	1	2277	2.4	0.0	191.4	0.50
PLA2G2A	1	933	2	2276	1.2	0.0	23.5	1.00
POLE	1	933	3	2275	0.8	0.0	10.1	1.00
ATRIP	1	933	4	2274	0.6	0.0	6.2	1.00
DNAJC21	1	933	4	2274	0.6	0.0	6.2	1.00
ERCC3	1	933	10	2268	0.2	0.0	1.7	0.19
ERCC6	1	933	0	2278	n.a.			0.29
FAAP24	1	933	0	2278	n.a.			0.29
APEX1	0	934	5	2273	0.0	0.0	2.7	0.33
CASP8	1	933	1	2277	2.4	0.0	191.4	0.50
DMC1	1	933	1	2277	2.4	0.0	191.4	0.50
FANCE	1	933	1	2277	2.4	0.0	191.4	0.50
FLCN	1	933	1	2277	2.4	0.0	191.4	0.50
RAD1	1	933	1	2277	2.4	0.0	191.4	0.50
SDHAF2	1	933	1	2277	2.4	0.0	191.4	0.50
XRCC4	1	933	1	2277	2.4	0.0	191.4	0.50
CWF19L2	1	933	2	2276	1.2	0.0	23.5	1.00
POLD1	1	933	2	2276	1.2	0.0	23.5	1.00
TLR4	1	933	2	2276	1.2	0.0	23.5	1.00
NCAM1	1	933	3	2275	0.8	0.0	10.1	1.00
PRF1	1	933	3	2275	0.8	0.0	10.1	1.00
TSHR	1	933	3	2275	0.8	0.0	10.1	1.00
LIG3	1	933	4	2274	0.6	0.0	6.2	1.00
RAD54B	1	933	5	2273	0.5	0.0	4.4	0.68
DCLRE1C	1	933	10	2268	0.2	0.0	1.7	0.19
MDC1	1	933	13	2265	0.2	0.0	1.2	0.080
BRAP	1	933	0	2278	n.a.			0.29
GRB7	1	933	0	2278	n.a.			0.29
HOXB13	1	933	0	2278	n.a.			0.29
CHEK1	1	933	0	2278	n.a.			0.29
LIG4	1	933	0	2278	n.a.			0.29
PTCH1	1	933	0	2278	n.a.			0.29
RAD9A	1	933	0	2278	n.a.			0.29
RUNX1	1	933	0	2278	n.a.			0.29
SBDS	1	933	0	2278	n.a.			0.29
TERF2	1	933	0	2278	n.a.			0.29
AIP	0	934	1	2277	0.0	0.0	95.0	1.00
FAM175A	0	934	1	2277	0.0	0.0	95.0	1.00
EPHX1	0	934	2	2276	0.0	0.0	13.0	1.00
RBBP8	0	934	2	2276	0.0	0.0	13.0	1.00
ERCC2	0	934	6	2272	0.0	0.0	2.1	0.19
RFC1	0	934	0	2278	n.a.			1.00
ATMIN	0	934	1	2277	0.0	0.0	95.0	1.00
BABAM1	0	934	1	2277	0.0	0.0	95.0	1.00
BRE	0	934	1	2277	0.0	0.0	95.0	1.00
CSNK1D	0	934	1	2277	0.0	0.0	95.0	1.00
CYLD	0	934	1	2277	0.0	0.0	95.0	1.00
DDB2	0	934	1	2277	0.0	0.0	95.0	1.00
ERCC4	0	934	1	2277	0.0	0.0	95.0	1.00
EXT2	0	934	1	2277	0.0	0.0	95.0	1.00
EYA2	0	934	1	2277	0.0	0.0	95.0	1.00
FANCL	0	934	1	2277	0.0	0.0	95.0	1.00
HUS1	0	934	1	2277	0.0	0.0	95.0	1.00
KCNJ5	0	934	1	2277	0.0	0.0	95.0	1.00
NELFB	0	934	1	2277	0.0	0.0	95.0	1.00
NFKBIZ	0	934	1	2277	0.0	0.0	95.0	1.00
NSD1	0	934	1	2277	0.0	0.0	95.0	1.00
POLB	0	934	1	2277	0.0	0.0	95.0	1.00
PRKDC	0	934	1	2277	0.0	0.0	95.0	1.00
PTTG2	0	934	1	2277	0.0	0.0	95.0	1.00
RHBDF2	0	934	1	2277	0.0	0.0	95.0	1.00
TCL1A	0	934	1	2277	0.0	0.0	95.0	1.00
TP53BP1	0	934	1	2277	0.0	0.0	95.0	1.00
TSC1	0	934	1	2277	0.0	0.0	95.0	1.00
UBE2V2	0	934	1	2277	0.0	0.0	95.0	1.00
XPC	0	934	1	2277	0.0	0.0	95.0	1.00
ZNF350	0	934	1	2277	0.0	0.0	95.0	1.00
CEP57	0	934	2	2276	0.0	0.0	13.0	1.00
FANCB	0	934	2	2276	0.0	0.0	13.0	1.00
MUTYH	0	934	2	2276	0.0	0.0	13.0	1.00
RFC1	0	934	2	2276	0.0	0.0	13.0	1.00
XRCC2	0	934	2	2276	0.0	0.0	13.0	1.00
XRCC3	0	934	2	2276	0.0	0.0	13.0	1.00
ESR2	0	934	3	2275	0.0	0.0	5.9	0.56
HNF1A	0	934	3	2275	0.0	0.0	5.9	0.56
MSH5	0	934	3	2275	0.0	0.0	5.9	0.56
PREX2	0	934	4	2274	0.0	0.0	3.7	0.33
FANCC	0	934	5	2273	0.0	0.0	2.7	0.33
SMAD4	0	934	5	2273	0.0	0.0	2.7	0.33
AURKA	0	934	9	2269	0.0	0.0	1.2	0.067

Table S6. Characteristics of 15 *PPM1D* mutation carriers. (The increasing age at OC diagnoses and the time of blood sampling from the last chemotherapy are displayed in a red-to-green gradient)

ID	Age at dg.	Center	Exon	c.	p.	WT reads	MUT reads	MAF	Genetic sampling	Last chemoth.	Sampling from last chemoth. (years)	Other Pathogenic mut.
3094	43.9	VFN	6	c.C1528T	p.Q510X	89	14	0,14	21-Apr-2016	15-Aug-2012	3.68	-
cz1475	56.0	VFN	6	c.1632dupC	p.G544fs	128	52	0,29	n.a.	n.a.	n.a.	-
306_16	59.0	MOU	6	c.1524delG	p.M508fs	129	35	0,21	n.a.	n.a.	n.a.	*EXO1*
2480	59.7	VFN	6	c.1449dupT	p.T483fs	106	18	0,15	24-Apr-2014	14-Mar-2007	7.11	-
1897	60.1	VFN	6	c.C1403G	p.S468X	69	13	0,16	12-Apr-2012	23-Jun-2009	2.80	-
CZE239	63.0	ANJ	6	c.C1372T	p.R458X	65	15	0,19	n.a.	n.a.	n.a.	-
2045	64.8	VFN	6	c.1529delA	p.Q510fs	84	52	0,38	n.a.	n.a.	n.a.	*BRCA2*
3120	64.8	VFN	6	c.1449delT	p.T483fs	178	33	0,16	n.a.	n.a.	n.a.	*BRCA2*
5263_17	65.0	GEN	1	c.325delG	p.G109fs	106	19	0,15	n.a.	n.a.	n.a.	*BRCA2*
			6	c.1286delG	p.R429fs	29	43	0,60				
1804	66.3	VFN	6	c.A1645T	p.K549X	76	12	0,14	8-Dec-2011	14-Sep-2005	6.23	-
4179	66.5	VFN	6	c.C1654T	p.R552X	106	22	0,17	14-Jun-2018	17-Jan-2018	0.41	-
10018_17	67.0	GEN	6	c.G1270T	p.E424X	51	9	0,15	n.a.	n.a.	n.a.	-
2915	67.2	VFN	6	c.1386_1387del	p.462_463del	49	25	0,34	3-Nov-2015	25-Jun-2015	0.36	*PALB2*
8395_17	70.0	GEN	6	c.1612delT	p.L538X	77	49	0,39	n.a.	n.a.	n.a.	-
1822	70.5	VFN	6	c.1607delG	p.R536fs	93	22	0,19	12-Jan-2012	14-Dec-2010	1.08	*PMS1*

Table S7. Clinicopathological characteristics of mutation carriers in *NAT1*, *PPM1D*, and *SHPRH* significantly associated with OC risk.

**Category**	**N patients**	***NAT1***	***PPM1D***	***SHPRH***	***Σ***	**%**
Age at diagnosis (y)						
30-39	152	4			4	2.63
40-49	282	2	1	1	4	1.42
50-59	360	3	3	1	7	1.94
60-69	328	3	9	3	15	4.57
>70	94	1	2		3	3.19
Personal history						
OC only	1043	9	11	3	23	2.21
OC & BC	210	2	2	1	5	2.38
OC & other cancer	78	2	2	1	5	6.41
Family history						
HBOC	345	2	4	1	7	2.03
HOC	119	2	1		3	2.52
Multiple cancer	182	1	1	1	3	1.65
None	588	6	9	3	18	3.06
n.a.	99	2			2	2.02
Histology						
HG serous	478	3	5	3	11	2.30
LG serous	85		1		1	1.18
Serous unspecified	165	2	4	1	7	4.24
Endometrioid	90		3		3	3.33
Clear cell	15	1			1	6.67
Other	90	1			1	1.11
Border-line	154	3			3	1.95
n.a.	213	3	2	1	6	2.82
Σ	*1333*	*13*	*16*	*5*	*34*	*2.55*
